# Pro-neurogenic effect of fluoxetine in the olfactory bulb is concomitant to improvements in social memory and depressive-like behavior of socially isolated mice

**DOI:** 10.1038/s41398-020-0701-5

**Published:** 2020-01-27

**Authors:** Leonardo O. Guarnieri, Ana Raquel Pereira-Caixeta, Daniel C. Medeiros, Nayara S. S. Aquino, Raphael E. Szawka, Eduardo M. A. M. Mendes, Márcio F. D. Moraes, Grace S. Pereira

**Affiliations:** 1 Núcleo de Neurociências, Universidade Federal de Minas Gerais, Belo Horizonte, Brazil; 2 Centro de Tecnologia e Pesquisa em Magneto Ressonância, Programa de Pós-Graduação em Engenharia Elétrica, Universidade Federal de Minas Gerais, Belo Horizonte, Brazil; 3 Laboratório de Endocrinologia, Departamento de Fisiologia e Biofísica, Instituto de Ciências Biológicas, Universidade Federal de Minas Gerais, Belo Horizonte, Brazil

**Keywords:** Long-term memory, Depression, Physiology

## Abstract

Although loneliness is a human experience, it can be estimated in laboratory animals deprived from physical contact with conspecifics. Rodents under social isolation (SI) tend to develop emotional distress and cognitive impairment. However, it is still to be determined whether those conditions present a common neural mechanism. Here, we conducted a series of behavioral, morphological, and neurochemical analyses in adult mice that underwent to 1 week of SI. We observed that SI mice display a depressive-like state that can be prevented by enriched environment, and the antidepressants fluoxetine (FLX) and desipramine (DES). Interestingly, chronic administration of FLX, but not DES, was able to counteract the deleterious effect of SI on social memory. We also analyzed cell proliferation, neurogenesis, and astrogenesis after the treatment with antidepressants. Our results showed that the olfactory bulb (OB) was the neurogenic niche with the highest increase in neurogenesis after the treatment with FLX. Considering that after FLX treatment social memory was rescued and depressive-like behavior decreased, we propose neurogenesis in the OB as a possible mechanism to unify the FLX ability to counteract the deleterious effect of SI.

## Introduction

Even though humans are naturally social animals, choosing to be alone can provide a positive state of satisfaction. Conversely, loneliness, characterized by being a non-temporary condition, is a subjective state with negative impacts on physical and mental health^1–3^. In fact, the consistent unpleasant experience of being alone can even predict depressive symptomatology^4–7^.

Depression is a mood disorder with heterogeneous symptoms and etiology (American Psychiatric Association, 1994). Despite the behavioral symptoms^6–8^, appetite and weight changes are common, though inconstant physiological markers for depression^8^. Anatomically, hippocampus and olfactory bulb (OB) are reduced in some cases of depression^9^. Furthermore, depression is usually associated with disruption of episodic memory^10,11^, such as impaired recollection, exacerbated memory for negative content^12^, and the weakening of positive memories^13,14^. Nevertheless, the mechanisms of memory disruption in depression are still poorly understood^15^.

The pharmacological treatment for depression includes fluoxetine, a selective serotonin (5-HT) reuptake inhibitor, and desipramine, a tricyclic antidepressant which inhibits the reuptake of norepinephrine (NE), but also 5-HT^16,17^. Lower concentrations of NE and 5-HT in the brain^18–20^ along with the effectiveness of antidepressant drugs^21,22^ support the monoaminergic hypothesis of depression.

Alternatively, neurogenesis has arisen as a pivotal mechanism in the pathogenesis of depression^23^. Unmedicated depressed adults presented less granule neurons in the anterior dentate gyrus (DG) when compared to healthy controls^24^, which agrees with reduced hippocampal volume findings observed in patients with major depression^25^. Accordingly, neurogenesis has been proposed by several independent researchers as one of the mechanisms for the antidepressant effect of fluoxetine^26,27^.

Evidence suggest that loneliness is not a solely human experience^28^. Objective loneliness can be induced in laboratory animals by depriving them from physical contact with conspecifics. Rearing mice on social isolation (SI) may induce depressive, anxious and aggressive behaviors later on life^29,30^ and it is usually used as an animal model for stress^31,32^. SI during adulthood, however, is less studied, although their behavioral effects has been shown to depend on SI duration^33^.

One emerging field that has been the focus of our research is to understand the effects of SI on a specific type of episodic memory named social recognition memory^23,34^. One week of SI is deleterious for the long-term maintenance of social memory in mice^35–37^, although did not affect other hippocampus-dependent memories^36^. Interestingly, enriched environment blunted the effect of SI on memory^36^ in a neurogenesis-dependent manner^38^. In fact, the formation of social recognition memory seems reliant on neurogenesis^39,40^.

Here we raised the hypothesis that SI is a condition that induces episodic memory impairment and depressive-like behaviors, allowing to investigate the mechanisms of memory disruption in depression. This work examines the behavioral, pharmacological, morphological, and neurochemical features of depression in isolated mice. We also tested the antidepressant potential of fluoxetine, desipramine, and the enriched environment in the SI model. Our study proposes neurogenesis as a core mechanism involved in the antidepressant and promnesic effects of fluoxetine and enriched environment.

## Material and methods

### Subjects

We used 164 adults (8–12 weeks of age) (30 females and 134 males) and 36 juveniles (21–35 days of age, only used as a social stimulus) Swiss mice. All animals were maintained in a climate-controlled environment (22 ± 2 °C, humidity at 55 ± 10%) under a 12 h light/dark cycle. All behavioral experiments were performed during the light phase. Both food and water were available ad libitum. The animals were randomly placed in one of the following groups: group housed in standard environment (CONTROL); group housed in enriched environment (CONTROL + ENRICHMENT); individually housed in standard environment (SOCIAL ISOLATION), and individually housed in enriched environment (SOCIAL ISOLATION + ENRICHMENT). Animals were maintained in polypropylene cages (28 cm × 17 cm × 12 cm), except the control + enrichment group that was maintained in bigger polypropylene cage (40 cm × 33 cm × 16 cm). The enriched environment was provided by the addition of ribbons, pieces of plastic, cardboard rolls and toys into the cage. Animals were maintained in each condition during at least 7 days.

All experiments were performed in compliance with the guidelines from the National Council for Animal Experimentation Control (CONCEA-BRAZIL). All protocols were approved by the Institutional Ethics Committee on the Use of Animals at the Universidade Federal de Minas Gerais (CEUA/UFMG) (no. 177/2014).

The investigator was blinded to the group allocation during the experiment and during assessing the outcoming results.

### Metabolic evaluation

Animals were weighted daily during 7 days. During the same period, the consumption of food and water was measured. All these measurements were taken at 9:00 a.m.

### Behavioral analysis

#### Forced-swimming test

Animals were placed individually in a glass cylinder (35 cm high, 24 cm in diameter) filled with water (28 °C) to the height of 14 cm. During 6 min, time of immobility was quantified^41,42^.

#### Tail suspension test

Mice were suspended by the tail and attached to a metal rod (50 cm height) for a period of 6 min. After 1 min habituation, total immobility time was scored. Immobility was considered as the absence of movements in the hindlimbs and forelimbs^43,44^.

#### Sucrose preference test

Animals were individually placed in cages containing two bottles, one filled with water and the other one with 3% sucrose solution. During the first 3 h, the bottles were weighed every 1 h. After, bottles were weighed 12, 24, and 48 h. Between each measure we changed the position of the bottle to avoid spatial cues. The preference for sucrose over water was calculated by the following formula: [sucrose consumed/(sucrose + water consumed)]. Results were expressed as the percentage of sucrose preference^45^.

#### Social recognition

Habituation phase consisted in introducing the adult mouse inside a clean cage (28 cm × 17 cm × 12 cm) containing an empty cylinder, with 60 evenly spaced holes on its walls, for a period of 30 min. During the last 5 min, a juvenile mouse was introduced into an identical cylinder, within its own cage. Training session (TR) lasted 5 min and consisted in replacing the empty cylinder by the one containing the juvenile mouse. Social exploration was scored every time the adult’s nose or whiskers were introduced in the cylinder’s holes. Test session (TT) was identical to TR, lasted 5 min and was performed either 1.5 h later, in case of testing short-term memory (STM) or 24 h after TR to test long-term memory (LTM). Results were expressed as social recognition index [time exploring the juvenile during TT/time exploring the juvenile during TR + TT]^40^.

#### Open field

An automated system (Actitrack v2.7.13.) was used to evaluate the locomotor activity in the open field. The apparatus consists of an acrylic arena (25 × 25 cm), coupled to two external infrared systems that monitor the total distance traveled by the animal during 5 min. The arena was cleaned with 70% alcohol after each animal^46^.

### Pharmacological treatment

Fluoxetine hydrochloride [(Sigma, St. Louis, MO) 30 and 45 mg/kg] and desipramine hydrochloride [Sigma, St. Louis, MO) 30 and 45 mg/kg] were diluted in saline and were administered intraperitoneally 20 min before the respective test (acute treatment) or daily during 7 days (chronic treatment).

### Magnetic resonance imaging

Animals were maintained anesthetized with isoflurane (3% induction and 1.5% maintenance) while inside the 4.7 T NMR system (Oxford). Imaging protocol consisted on the acquisition of T2-weighted coronal images (TR = 3000 ms, TE = 50 ms) with a total of 20 contiguous slices of 1 mm thickness (512 × 256 voxels)^40^.

Images were evaluated using masks for the OB and the hippocampus, constructed manually, using a tablet driver (Bamboo Tablet Driver, V5.2.5 WIN; WACOM Technology Corporation, USA) and MeVisLab software (MeVis Medical Solutions AG, Fraunhofer). Volumetric quantification was done using MatLab® scripts^47^.

### Quantification of serotonin (5-HT) and NE by high-performance liquid chromatography

OB, dorsal, and ventral hippocampus were homogenized in 450, 150, and 200 μL, respectively, of a solution containing 0.15 M perchloric acid, 0.1 mM EDTA, and 3,4-dihydroxybenzylamine (DHBA; Aldrich, Milwaukee, WI) as the internal standard. The homogenate was centrifuged for 20 min at 12,000*g*. Protein content was determined from the pellet by the Bradford method^48^. In the supernatant, concentrations of NE and serotonin (5-HT) were determined by high-performance liquid chromatography (HPLC) with electrochemical detection. Briefly, chromatographic separation was performed with a C18 column (Purospher Star, 5 μm, 250 × 4 mm; Merck Darmstadt, Germany), preceded by pre-column C18 (Lichrospher, 5 μm, 4 × 4 mm; Merck). The mobile phase consisted of 100 mM NaH_2_PO_4_, 10 mM NaCl, 0.1 mM EDTA, 0.38 mM sodium octanesulfonic acid, 10% methanol, and pH 3.5. The flow of the HPLC pump was adjusted to 1.0 mL/min and the potential of the electrochemical detector, 0.4 V (Decade II, VT-03 electrochemical flow cell; Antec Leyden, The Netherlands). Chromatographic data were analyzed using Class-VP software (Shimadzu, Kyoto, Japan). NE and 5-HT were identified according to their elution time and quantified using calibration curves by the internal standard method (DHBA). The intra-assay coefficient of variation was less than 5% for all measured compounds. NE and serotonin levels were considered to reflect neurotransmitter stores in the synaptic vesicles^49^.

### BrdU administration

Bromodeoxyuridine (BrdU; Sigma) was dissolved in 0.9% NaCl and administered intraperitoneally at 75 mg/kg, once a day for a total of 7 days^38–40^.

### Immunofluorescence

Anesthetized mice (80 mg/kg ketamine and 10 mg/kg xylazine) were submitted to transcardiac perfusion with 0.01 M phosphate-buffered saline (PBS) and subsequently 2% paraformaldehyde (PFA). Brains were removed, fixed overnight in 4% PFA, and kept in a 30% sucrose solution at 4 °C during 3 days. Coronal 40-μm brain sections were sliced through a cryostat and stored at −22 °C in PBSAF cryoprotectant solution (PBS, 20% sucrose, 15% ethylene glycol, 0.05% NaN_3_). For each animal, we selected as many as possible OB slices, six slices of dorsal hippocampus (1.70 mm to −2.30 mm from Bregma) and six slices of ventral hippocampus (−3.16 to −3.52 mm from Bregma)^50^. Slices were washed sequentially in PBS, PBST, and 0.9% NaCl. Subsequently, slices were incubated at 37 °C in 2 M HCl for 10 min and 3 M HCl for 30 min, washed in 0.1 M borate buffer. Slices were placed for 1 h 30 min in 5% normal goat serum and incubated for 72 h at 4 °C with either one of the following combinations: anti-BrdU (1:800; Abcam) and anti-NeuN (1:500; Millipore) or anti-BrdU (1:800; Abcam) and anti-GFAP (1:1000; Abcam), anti-BrdU marked newborn cells, anti-NeuN labeled neurons, and anti-GFAP marked astrocyte cells. After, slices were washed in PBS and incubated for 90 min at room temperature with Alexa Fluor 488 (1:400, Invitrogen) and Alexa Fluor 568 (1:400, Invitrogen), washed in PBS, and mounted. Slices were fixed in Vectashield (Vector Laboratories)^39,40^.

### Image acquisition and analysis

Quantification of BrdU^+^/NeuN^+^ and BrDU^+^/GFAP^+^ cells was achieved by counting the number of positive double-labeled cells using a ×40 objective of an epifluorescence microscope (Zeiss) and Axiovision 4.8 software. Cells were considered double-labeled when colabeling with relevant morphology was seen throughout the extent of the cells and viewed in *x*–*y*, *x*–*z,* and *y*–*z* cross-sections produced by orthogonal reconstructions from z-series. Cells were counted bilaterally, one by one, using ImageJ Software (NIH, USA). The experimenter counted the cells blind to the condition. The exposure time for each filter was determined through the pixel saturation histogram, and the maximum possible number of pixels below the saturation limit was always used^39,40^.

Cell density was calculated using the DAPI immunostaining described above. Slice photos were acquired using an epifluorescence microscope (Zeiss) and Axiovision 4.8 software at ×40 magnification. Each hippocampal region (CA1, CA3, and DG) and the internal and external granular layers of the OB were quantified within a rectangular area with a size between 2867 and 3959 μm^2^. The measurement of the area and counting the number of cells were performed using ImageJ Software (NIH, USA). All density results were expressed as the cell density per mm^2^.

### Statistical analysis

Sample size was estimated using the following formula: *n* = *S*^2^/(*μA* − *μ*0)^2^ × (*tα* + *Uβ*)^2^. Data were expressed as the mean ± standard error of mean (SEM) with the exception of magentic resonance imaging (MRI) analysis data, which were expressed in median ± interquartile range. Statistical analyses were performed using *Graph Pad Prism 7* software. Two-way ANOVA followed by Bonferroni’s multiple comparison test was used in social memory, forced-swimming test (FST), tail suspension test (TST), immunofluorescence, and % of neurogenesis. Data from social memory were also analyzed by one-sample *t*-test, with 0.5 as the hypothetical value. Two-way repeated measures ANOVA followed by Bonferroni’s multiple comparison test was used in sucrose preference and weight gain. Other results were analyzed by unpaired Student *t*-test.

## Results

### SI does not compromise social recognition memory in female mice

**Fig. 1:**
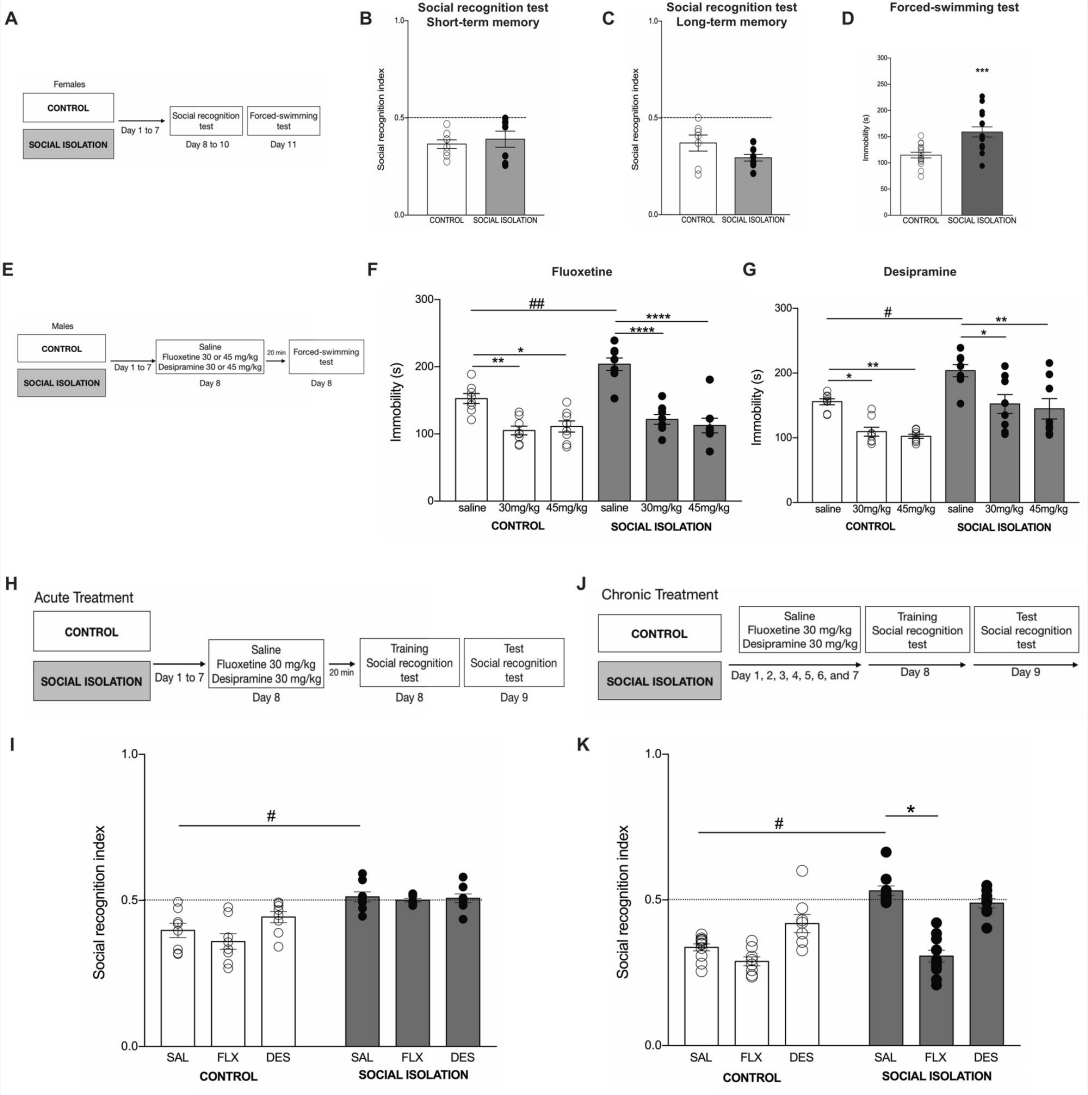
Social isolation (SI) did not affect social recognition memory in female mice. **a** Female mice were maintained in groups (control) or isolated (social isolation) during 7 days. Thereafter, animals were submitted to social recognition test for short (STM) and long-term memory (LTM) as well as forced-swimming test (FST). **b** Both groups presented STM and **c** LTM, and no difference between groups were detected. **d** SI increased immobility in female mice (***p < 0.001). **e** Male mice were maintained in groups (control) or isolated (social isolation) during 7 days. At day 8, animals received a single injection of saline (SAL), fluoxetine (FLX), or desipramine (DES) and 20 min after were exposed to forced-swimming test (FST). **f** Male mice presented an increase in immobility (depressive-like behavior) after social isolation (^##^*p* < 0.01). Fluoxetine, in both doses, decreased immobility in control and SI groups (*p < 0.05; **p < 0.01, and ****p < 0.0001). **g** Male mice presented an increase in immobility (depressive-like behavior) after social isolation (^#^*p* < 0.05). Desipramine, in both doses, decreased immobility in control and SI groups (*p < 0.05; **p < 0.01). **h** Male mice were maintained in groups (control) or isolated (social isolation) during 7 days. At day 8, animals received a single injection of SAL, FLX, or DES and 20 min after were exposed to the training session of the social recognition test. Twenty-four hours later, the social memory was tested. Results are presented as social recognition index [time exploring the juvenile during TT/time exploring the juvenile during TR + TT]. **i** SI mice differ from control (^#^*p* < 0.05) and acute treatment with antidepressants had no effect. **j** Male mice were maintained in groups (control) or isolated (social isolation) during 7 days. Every day, animals received a single injection of SAL, FLX, or DES. At day 8, the training session of the social recognition test was performed. Twenty-four hours later, the social memory was tested. Results are presented as social recognition index [time exploring the juvenile during TT/time exploring the juvenile during TR + TT]. **k** SI mice differ from control (^#^*p* < 0.05) and chronic treatment with FLX (*p < 0.05) rescued the social memory deficit of SI mice. Data are presented as mean ± SE.

There is evidence suggesting that SI affects males and females differentially^51^. Therefore, before assessing whether the social memory deficit caused by SI in male mice^36–38^ is accompanied by depressive-like behavior, we tested female mice in the social recognition task after 1 week of SI. We conducted experiments (Fig. 1a) to test short (STM, Fig. 1b) and long-term (LTM, Fig. 1c) social memory in female mice. We chose to test STM because it was the first time we tested females, and we know that SI did not compromise STM in males^36–38^. In the STM, there was no difference between groups (*t*_(13)_ = 0.5, *p* = 0.58). Additionally, both groups explored less the juvenile during the test (one-sample *t*-test: control: *t*_(7)_ = 6, *p* = 0.0005; social isolation: *t*_(6)_ = 2.6, *p* = 0.03). Similar results were observed for LTM. No difference between groups was detected (*t*_(13)_ = 1.7, *p* = 0.1). However, control and social isolated female mice explored less the juvenile during testing (one-sample *t*-test: control: *t*_(6)_ = 3.11, *p* = 0.02; social isolation: *t*_(7)_ = 11.8, *p* < 0.0001). Taken together, our results indicate that 1 week of SI did not affect social memory in female Swiss mice. We also tested females in the forced swim test (FST) and found that 1 week of SI induced depressive-like behavior in females (Fig. 1d: *t*_(28)_ = 3.9, *p* = 0.0005), as expected. However, in order to pursuit our initial question, which is to investigate whether the memory deficit and depressive behavior present a common neural mechanism in the context of SI, we conducted the experiments using male, since SI did not affect social memory in female mice.

### Depressive-like behavior induced by SI is rescued by the acute treatment with fluoxetine or desipramine

Thus, we evaluated the effect of SI on depressive-like behavior in male mice and also the effect of antidepressants (Fig. 1e). Two-way ANOVA followed by multicomparison test showed higher immobility time in SI mice and that fluoxetine (FLX) prevented this behavior to happen (Fig. 1f; Interaction: *F*_(2,42)_ = 4.5, *p* = 0.01; House condition: *F*_(1,42)_ = 11.2, *p* < 0.001; Treatment: *F*_(2,42)_ = 40.6, *p* < 0.0001). Similar results were observed for desipramine (DES) (Fig. 1g; Interaction: *F*_(2,42)_ = 0.04, *p* = 0.95; House condition: *F*_(1,42)_ = 28, *p* < 0.0001; Treatment: *F*_(2,42)_ = 17.7, *p* < 0.0001). Furthermore, SI effect on FST may not be attributed to locomotor activity, since SI and control groups behaved similarly in the open field (Control: 1844 ± 482.1 cm; Social isolation: 1810 ± 345.6 cm; unpaired *t*-test: *t*_(14)_ = 0.16, *p* = 0.87).

### Long-term social memory impairment caused by SI is rescued by chronic treatment with fluoxetine

Given that SI induces depressive-like behavior and social recognition memory deficit, we raised the question as to whether these two effects are related. Thus, we tested the ability of antidepressants to rescue the social recognition memory impaired by SI **(**Fig. 1h). The acute treatment with fluoxetine (FLX) or desipramine (DES) **(**Fig. 1i**)** produced a main effect of house condition (*F*_(1,42)_ = 46, *p* < 0.0001), but not of treatment (*F*_(2,42)_ = 2.6, *p* = 0.08) or interaction between factors (*F*_(2,42)_ = 2, *p* = 0.1). Post hoc analysis indicated a difference between control and SI treated with saline, confirming our previous results, but no effect of antidepressants was observed. Complementary analysis with one-sample *t*-test confirmed that the social recognition index of SI mice did not differ from chance (Saline: *t*_(7)_ = 0.7, *p* = 0.49; FLX: *t*_(7)_ = 0.2, *p* = 0.8; DES: *t*_(7)_ = 0.4, *p* = 0.6). And as expected, all control groups showed a social recognition index fitting a regular social memory (Saline: *t*_(7)_ = 4.1, *p* = 0.004; FLX: *t*_(7)_ = 5.2, *p* = 0.001; DES: *t*_(7)_ = 3, *p* = 0.01). Therefore, acute treatment with FLX or DES did not rescue the memory deficit of SI mice.

Next, we verified whether a chronic treatment would be efficient in rescuing the social recognition memory deficit in SI mice (Fig. 1j). Two-way ANOVA revealed interaction between factors (*F*_(2,50)_ = 12.3, *p* < 0.0001) and a main effect for house condition (*F*_(1,50)_ = 36.8, *p* < 0.0001) and treatment (*F*_(2,50)_ = 39.6, *p* < 0.0001). Then again, Bonferroni’s post-test analysis showed a difference between control and SI mice. Additionally, SI mice treated with FLX presented a lower social recognition index compared to the SI-saline group (Fig. 1k), indicating they have LTM. As expected, all control groups showed a social recognition index fitting a regular social memory (Saline: *t*_(10)_ = 13.6, *p* < 0.0001; FLX: *t*_(7)_ = 13.4, *p* < 0.0001; DES: *t*_(7)_ = 2.6, *p* = 0.03). SI mice presented the memory deficit (Saline: *t*_(9)_ = 1.9, *p* = 0.08). Interestingly, FLX (*t*_(10)_ = 9.7, *p* < 0.0001), but not DES (*t*_(7)_ = 0.6, *p* = 0.5) treated SI mice showed a recognition index different from chance.

We also evaluated the effect of chronic treatment with FLX and DES in FST [Data showed as mean±SEM: control (*n* = 11, 159.6 ± 16.6 s); control + FLX (*n* = 8, 101.3 ± 14.9 s); control + DES (*n* = 8, 109.6 ± 16.5 s); SI (*n* = 10, 201.9 ± 21.2 s); SI + FLX (*n* = 11, 109.6 ± 11.4 s); SI + DES (*n* = 8, 142.3 ± 31 s)]. Two-way ANOVA showed an interaction (*F*_(2,50)_ = 3.9, *p* = 0.02) between factors and a main effect of house condition (*F*_(1,50)_ = 28.7, *p* < 0.0001) and treatment (*F*_(2,50)_ = 81.7, *p* < 0.0001). Multicomparison test confirmed the antidepressant effect of FLX and DES in control animals (*p* < 0.0001) and showed that FLX and DES decreased immobility in SI mice (*p* < 0.0001). We also reproduced the depressive-like behavior in SI mice (*p* < 0.0001).

### Enriched environment prevents the depressive-like state promoted by SI

We have shown that SI induced depressive-like behaviors and impaired social recognition memory. Antidepressants recovered the depressive-like behavior, but only the chronic treatment with fluoxetine was able to counteract SI effect on social memory. As we showed before^38^ that enriched environment (EE) rescues the deleterious effect of SI on social memory, here we tested whether EE would also act as antidepressant.

**Fig. 2:**
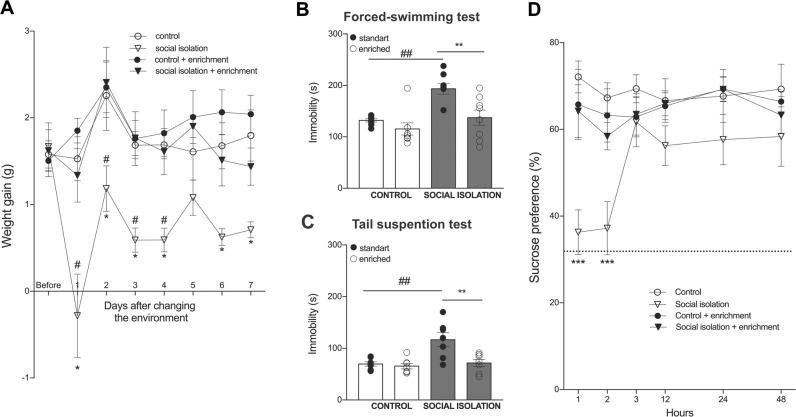
Antidepressant effect of enriched environment. **a** Loss of weight caused by social isolation was prevented by enriched enrichment. *p* < 0.05 indicates difference within the group and ^#^*p* < 0.05 indicates difference between groups. **b**, **c** Depressive-like behavior in social isolated mice was prevented by the enriched environment (* and ^##^*p* < 0.01). **d** Social isolated mice avoid sucrose solution in the first two hours of exposure (***p < 0.001), while enriched environment prevented such behavior. Data are presented as mean ± SE.

Appetite and weight changes are common, though inconstant physiological markers for depression in humans^12^. In our model, we found a profound weight loss along the 7 days of isolation that was completely blocked by the EE (Interaction: *F*_(21,196)_ = 1.3, *p* = 0.1; Time: *F*_(7,196)_ = 4.1, *p* = 0.0003; House condition: *F*_(3,28)_ = 33.7, *p* < 0.0001) (Fig. 2a). As showed in Fig. 1, SI mice spend more time immobile during the FST (Fig. 2b). Interestingly, EE was antidepressant, but only for SI mice (Interaction: *F*_(1,28)_ = 3.2, *p* = 0.08; House condition: *F*_(1,28)_ = 11, *p* = 0.002; Social Stimulus: *F*_(1,28)_ = 14.5, *p* = 0.0007). We also tested animals in the TST and found similar results. SI increased immobility, while EE prevented such effect, without changing behavior of control animals (Interaction: *F*_(1,24)_ = 6.1, *p* = 0.02; House condition: *F*_(1,24)_ = 8.8, *p* = 0.006; Social stimulus: *F*_(1,24)_ = 10.2, *p* = 0.003) (Fig. 2c).

Decreased ability to experience pleasure is another core symptom of depression^12,52,53^. Therefore, we tested whether SI would affect hedonic behavior in sucrose preference test. Socially isolated mice did not preferer sucrose in the first 2 h of testing, while EE normalize such behavior (Interaction: *F*_(15,40)_ = 2.3, *p* = 0.004; Time: *F*_(5,140)_ = 3.6, *p* = 0.004; House condition: *F*_(3,28)_ = 4.7, *p* = 0.008) (Fig. 2d).

### Decreased NE levels and reduced OB volume in socially isolated mice

**Fig. 3:**
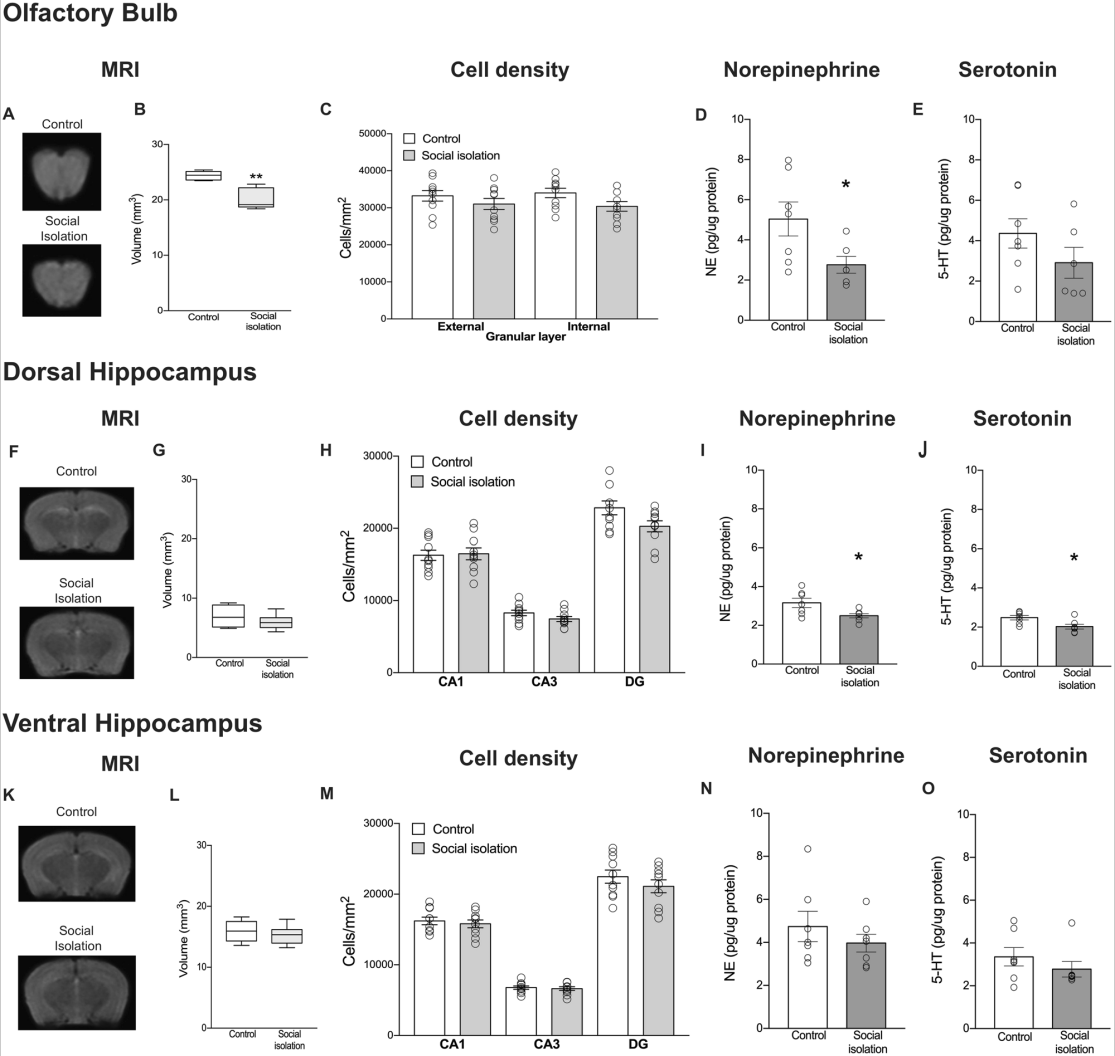
Social isolation decreased (**a**, **b**) the OB volume (**p < 0.01), did not affect (**c**) cell density, diminished (**d**) norepinephrine (NE) concentration (**p < 0.01), while no effect on (**e**) serotonin (5-HT) was observed. No effect of social isolation was observed in **f**, **g** dorsal hippocampus volume or **h** cell density, though **e** NE (*p < 0.05) and **f** 5-HT decreased (*p < 0.05). No effect of social isolation was observed in ventral hippocampus **k, l** volume, **m** cell density, **n** NE, and **o** 5-HT levels. The HPLC and cell density results are presented as mean±SE. MRI results are expressed in median ± interquartile range.

To further characterize the SI effect on brain function, we sought to investigate whether one week of SI in adulthood would alter morphological and neurochemical features related to depressive behaviors. One interesting aspect about acute major depressive patients is the presence of a smaller OB volume^13^, which is in accordance with the bulbectomy in rodents being proposed as an animal model for depression^54,55^. To address this question, we submitted mice to MRI after SI (Fig. 3a**)**. We found that mice that underwent to SI have reduced OBs compared to control (Fig. 3b: *t*_(8)_ = 4.5, *p* = 0.002). We also estimated cell density, but found no difference between groups in both external (*t*_(18)_ = 1.0, *p* = 0.2) and internal (*t*_(18)_ = 1.9, *p* = 0.06) granular layer of the OB (Fig. 3c).

Neurochemical unbalance may also be present in humans^18,19^ and rodents^21,35–37^ under a depressive state. Particularly serotonin (5-HT) and NE levels may be disturbed in those situations^18,20,36^. Thus, we verified whether SI would alter NE and 5-HT levels in OB homogenates. SI decreased NE levels in the OB (Fig. 3d: *t*_(11)_ = 2.2, *p* = 0.04), while no effect of SI was observed in 5-HT levels (Fig. 3e: *t*_(11)_ = 1.3, *p* = 0.1).

### SI reduces NE and serotonin in the dorsal hippocampus

In addition to the OB, the hippocampus is also sensitive to the effects of chronic depression^56,57^. Therefore, we investigated whether the SI would affect the volume, as well as the NE and 5-HT levels in dorsal (dHIP) and ventral hippocampus (vHIP).

The MRI analysis (Fig. 3f**)** showed no difference between groups regarding the volume of the dHIP (Fig. 3g: *t*_(10)_ = 0.9, *p* = 0.3). Cell density (Fig. 3h) in CA1 (*t*_(18)_ = 0.1, *p* = 0.8) and CA3 (*t*_(18)_ = 1.6, *p* = 0.1) was similar between groups, though in the DG there was a tendency for SI to reduce cell density (*t*_(18)_ = 2.0, *p* = 0.05). Interestingly, SI decreased NE (Fig. 3i: *t*_(12)_ = 2.5, *p* = 0.02) and 5-HT (Fig. 3j: *t*_(12)_ = 2.7, *p* = 0.01) in the dHIP.

In vHIP (Fig. 3k) we also did not find difference between groups regarding the volume (Fig. 3l: *t*_(10)_ = 0.6, *p* = 0.5). Furthermore, no difference between groups was observed in cell density measured in CA1 (*t*_(18)_ = 0.5, *p* = 0.5), CA3 (*t*_(18)_ = 0.4, *p* = 0.6), and DG (*t*_(18)_ = 1.0, *p* = 0.3) (Fig. 3m). In addition, neither the NE (Fig. 3n: *t*_(12)_ = 0.9, *p* = 0.3) nor the 5-HT levels (Fig. 3o: *t*_(10)_ = 1, *p* = 0.3) were altered after SI.

### Fluoxetine increased cell proliferation in the dHIP of SI mice

Previous studies from our group showed that enriched environment rescued the memory deficit of SI mice in a neurogenesis-dependent manner^38^. Antidepressants, such as fluoxetine (FLX), are well known to modulate neurogenesis^58–60^. Thus, we verified the effect of antidepressants in combination with SI on neurogenesis.

**Fig. 4:**
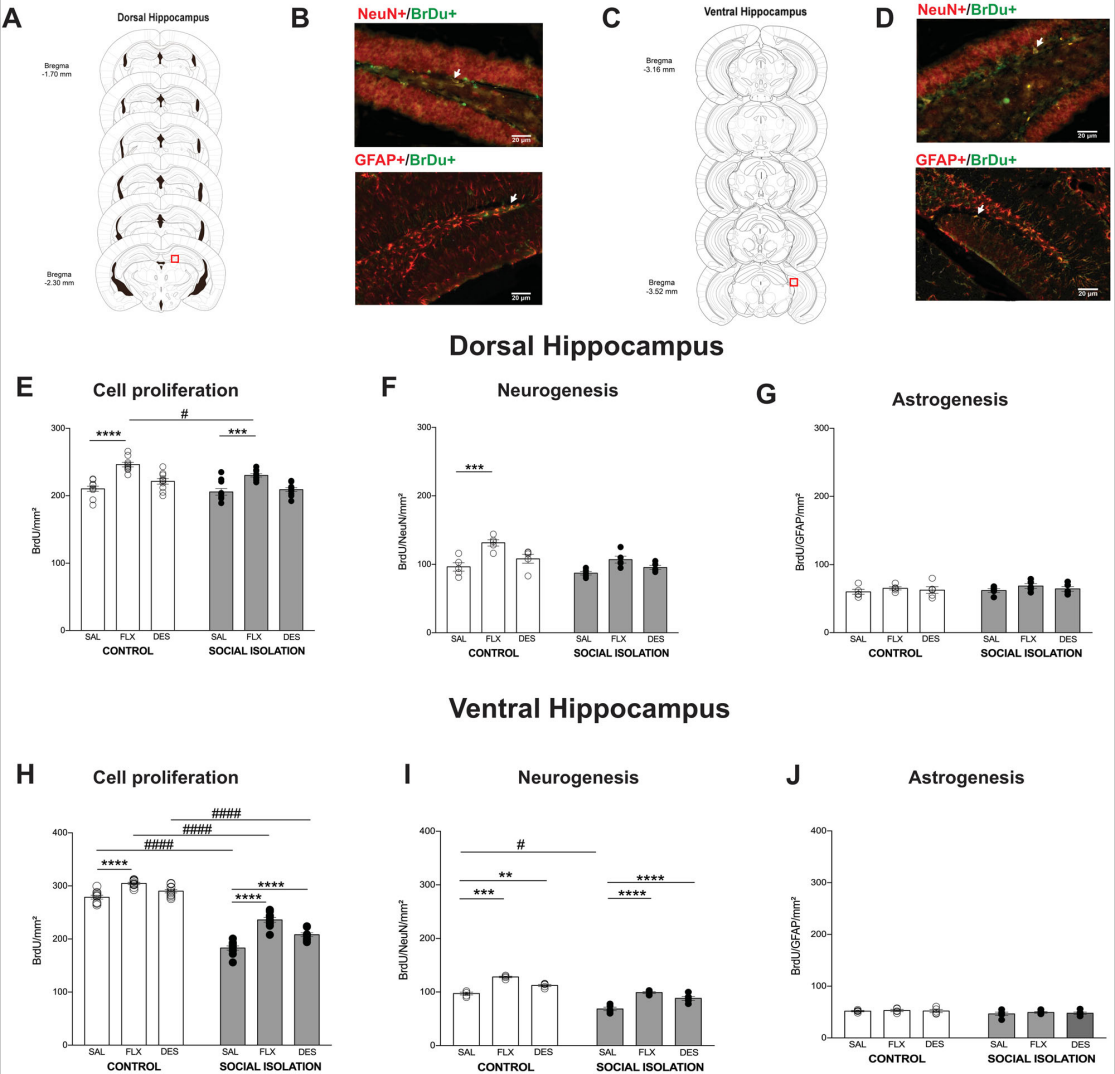
The effect of chronic treatment with antidepressants on **a**, **b** dHIP and **c**, **d** vHIP neurogenesis. **e** Cell proliferation, **f** neurogenesis, and **g** astrogenesis in dHIP. **h** Cell proliferation, **i** neurogenesis, and **j** astrogenesis in vHIP (** and ^#^*p* < 0.01; ***p < 0.001; **** and ^####^*p* < 0.0001). Data are presented as mean ± SE.

We analyzed dHIP (Fig. 4a, b) and vHIP (Fig. 4c, d). In dHIP, only FLX increased cell proliferation in both control and SI mice, even though its effect was less expressive in SI mice (Interaction: *F*_(2,54)_ = 1.2, *p* = 0.28; Treatment: *F*_(2,54)_ = 36.9, *p* < 0.0001; House condition: *F*_(1,54)_ = 13.3, *p* = 0.0006) (Fig. 4e). As predicted, FLX increased neurogenesis (Fig. 4f); however, no effect on SI mice was observed (Interaction: *F*_(2,24)_ = 1.2, *p* = 0.29; Treatment: *F*_(2,24)_ = 15.9, *p* < 0.0001; House condition: *F*_(1,24)_ = 14.7, *p* = 0.0008). Astrogenesis (Fig. 4g) was unchanged in all conditions evaluated (Interaction: *F*_(2,24)_ = 0.03, *p* = 0.9; Treatment: *F*_(2,24)_ = 1.3, *p* = 0.26; House condition: *F*_(1,24)_ = 0.6, *p* = 0.4).

### SI decreased neurogenesis in the ventral hippocampus

Motivated by vHIP role on social behavior^61–64^ and depression^65–67^ we investigated whether SI would affect cell proliferation, neurogenesis, and astrogenesis in this area. SI decreased cell proliferation, while FLX and DES increased it. In control animals, only FLX was effective in increasing cell proliferation (Interaction: *F*_(2,54)_ = 7.2, *p* = 0.001; Treatment: *F*_(2,54)_ = 61.9, *p* < 0.0001; House condition: *F*_(1,54)_ = 801.2, *p* < 0.0001) (Fig. 4h). Both antidepressants increased neurogenesis in control mice (Fig. 4i). Similar pattern was observed in SI mice, though the neurogenesis level was lower (Interaction: *F*_(2,24)_ = 0.63, *p* = 0.54; Treatment: *F*_(2,24)_ = 71.8, *p* < 0.0001; House condition: *F*_(1,24)_ = 168.9, *p* < 0.0001). In general, astrogenesis was decreased in SI mice, though post hoc analysis failed to detect statistical difference between groups (Interaction: *F*_(2,24)_ = 0.06, *p* = 0.9; Treatment: *F*_(2,24)_ = 0.3, *p* = 0.6; House condition: *F*_(1,24)_ = 5.1, *p* = 0.03) (Fig. 4j).

### Fluoxetine rescued neurogenesis in OB, suppressed by SI

**Fig. 5:**
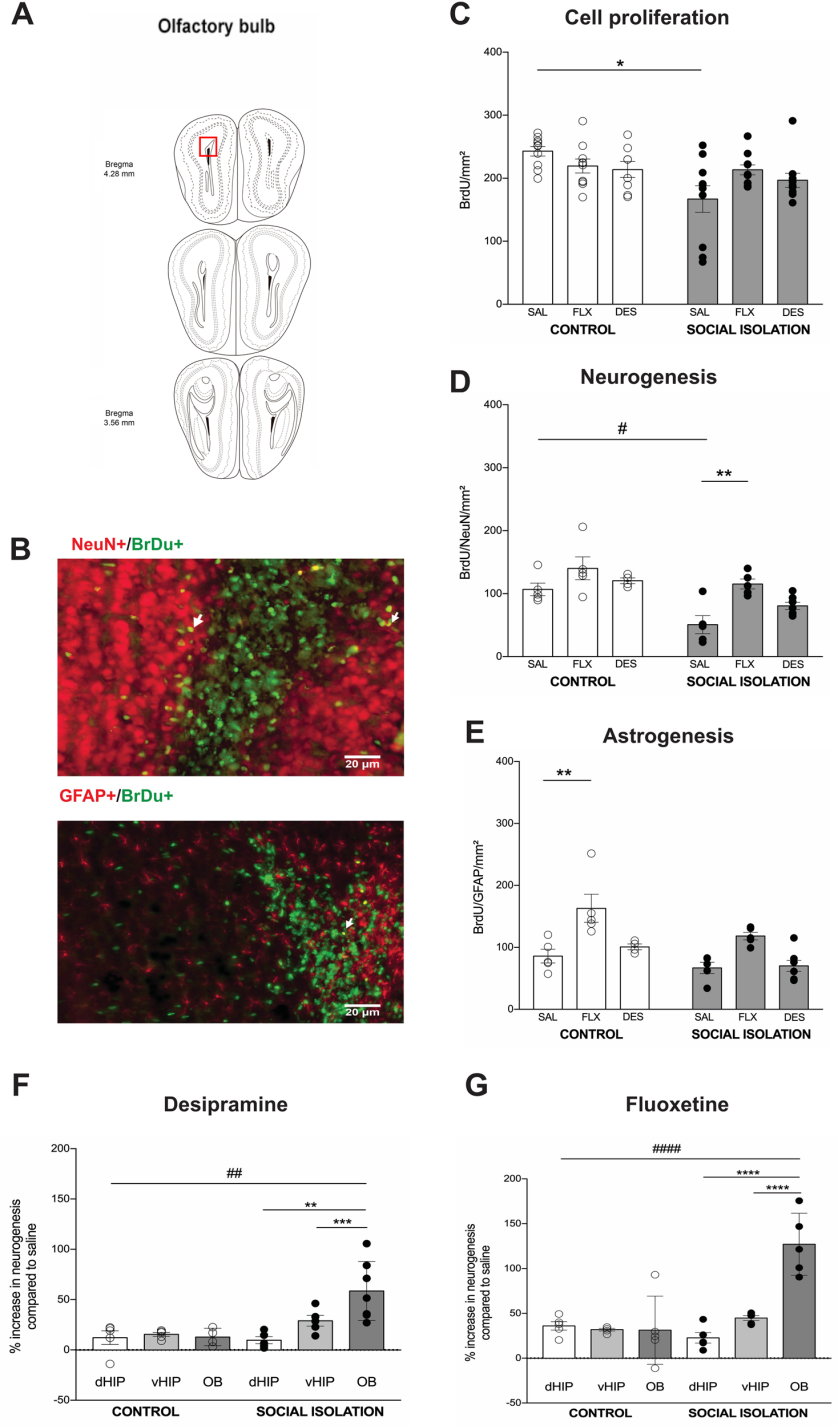
The effect of chronic treatment with antidepressants on **a**, **b** olfactory bulb neurogenesis. **d** Cell proliferation, **e** neurogenesis, and **f** astrogenesis. Olfactory bulb (OB) responded better than dorsal (dHIP) and ventral (vHIP) hippocampus to **g** desipramine and **h** fluoxetine chronic treatment, but only in animals under social isolation (* and ^#^*p* < 0.05; ** and ^##^*p* < 0.01; ***p < 0.001; **** and ^####^*p* < 0.0001). All data are presented as mean ± SE.

Finally, we evaluated the effect of antidepressants and SI on the OB (Fig. 5a) neurogenesis, specifically the granular layer (Fig. 5b). SI decreased cell proliferation (Interaction: *F*_(2,56)_ = 3.2, *p* = 0.04; Treatment: *F*_(2,56)_ = 0.2, *p* = 0.7; House condition: *F*_(1,56)_ = 3.9, *p* = 0.05) (Fig. 5c). Similar result was observed for neurogenesis, adding the fact that FLX blocked the SI effect (Interaction: *F*_(2,25)_ = 0.9, *p* = 0.9; Treatment: *F*_(2,25)_ = 9.6, *p* = 0.0008; House condition: *F*_(1,25)_ = 19.5, *p* = 0.0002) (Fig. 5d). Interestingly, differently from the hippocampus, FLX increased astrogenesis, though statistical significance was reached only for control animals (Interaction: *F*_(2,25)_ = 0.5, *p* = 0.5; Treatment: *F*_(2,25)_ = 16.1, *p* < 0.0001; House condition: *F*_(1,25)_ = 10, *p* = 0.004) (Fig. 5e).

### Under SI, fluoxetine increases region-dependent neurogenesis

To verify whether a neurogenesis niche would be more sensitive to antidepressants than others, and consequently estimate a possible relationship between the neurogenesis and the behavioral output, we calculated the percentage of increase in neurogenesis compared to saline in both control and social isolated animals. Figure 5f depicts the DES results. There was interaction (house condition and brain region, *F*_(2,25)_ = 5.3, *p* = 0.01), as well as main effect for both factors (House condition: *F*_(1,25)_ = 9.3, *p* = 0.005; Brain region: *F*_(2,25)_ = 5.4, *p* = 0.01). Post hoc analysis showed that in SI mice, neurogenesis in the OB was higher than in the dHIP and vHIP. Furthermore, a difference between control and SI was observed only in the OB. No difference between areas was observed in the control group. Regarding FLX, two-way ANOVA revealed interaction (house condition and brain region, *F*_(2,24)_ = 16.3, *p* < 0.0001), as well as main effect for both factors (House condition: *F*_(1,24)_ = 15.2, *p* < 0.0001; Brain region: *F*_(2,24)_ = 14.1, *p* < 0.0001) (Fig. 5g). Multiple comparison test detected that the increase in neurogenesis in the OB of SI mice was higher compared to dHIP and vHIP. It was also higher compared to OB from control animals. As for DES, no difference between areas was observed in the control group.

## Discussion

The ubiquitous manifestation of memory failures in depressed patients suggests that some level of overlap may exist in the neural mechanisms of depression and episodic memories. Yet, current understanding about the neural mechanisms of memory disruption in depression is limited. Here, we showed that social recognition memory deficit and depressant phenotype emerge concomitantly when adult Swiss mice are socially isolated for 7 days, suiting an appropriate model to investigate the mechanisms of memory disruption in depression.

It has been evidenced that social contact during specific phases of development is determinant for adult rodents to maintain stable emotional behaviors^68–70^. Here, we complemented this point of view by showing that Swiss mice behave according to a depressive phenotype, after being isolated from co-specifics for 7 days in the adulthood (SI mice).

C57/BL6 mice are usually more resistant to models that induce depressive-like behaviors^71,72^. On contrary, 1 week of SI is sufficient to induce social memory deficits in both C57/BL6 (ref. ^38^) and Swiss mice^38^. We chose to focus on Swiss mice in the present study to keep unified the SI duration to induce both memory deficit and depressive-like behavior. We found that adult SI mice gained less weight, as it was observed in juvenile Swiss mice submitted to isolation^70^. In contrast, no change in body weight was observed in C57/BL6 mice isolated for 28 days in the adulthood^73^. Intriguingly, appetite and weight changes are often the most discriminating symptoms to diagnose human depression subtypes^74,75^. In fact, the symptoms of depression disorders include significant weight loss or weight gain^76^. Supporting this apparent paradox, it was showed an opposite activation of the mid-insula from depressed subjects with appetite-increased and appetite-decreased, in response to food images^77^. Therefore, our study proposes that 7 days of SI in Swiss mice represent an ideal animal model to better comprehend the neural basis of weight loss in depression.

Lower concentrations of NE and serotonin (5-HT) in the brain^18–20,23–25^ along with the effectiveness of antidepressant drugs^21,22^ support the monoaminergic hypothesis of depression. Here, SI reduced NE in the OB and the dorsal hippocampus (dHIP), and 5-HT in the dHIP. Furthermore, fluoxetine (30 and 45 mg/kg) and desipramine (30 and 45 mg/kg) decreased immobility time of SI mice in the FST. Altogether, these results suggest that a monoaminergic unbalance may play a role on the depressive behaviors caused by SI.

Alternatively, the neural plasticity theory of depression proposes neurogenesis as a key element in the pathogenesis of depression^78^. In other words, neurogenesis in the hippocampus decreases in depression, while antidepressants effectiveness depends on neurogenesis upregulation^79^. Here, SI decreased cell proliferation and neurogenesis in the vHIP, while both antidepressants restored neurogenesis in this very same region. Interestingly, no changes in NE and 5-HT were observed in vHIP. Therefore, vHIP presents as the ideal neural substrate for the neural plasticity theory of depression in the context of ST. In fact, this assumption agrees with the notion that dHIP and vHIP functions are associated with memory and affective behaviors, respectively^80–82^.

DES and FLX have distinct mechanism of action, while the former inhibits NE reuptake^83,84^, the second selectively inhibits serotonin reuptake^85,86^. FLX effect in increasing neurogenesis is well grounded in the literature^87–89^. Interestingly, one study that measured neurogenesis based on specific cell types showed that FLX effects depend on the dorsoventral axis of the hippocampus^27^. However, in the present study we used a general neurogenesis marker and found a similar effect of FLX in dHIP and vHIP neurogenesis.

Compared to FLX, DES effect on neurogenesis has been less explored. It was showed that DES is effective in increasing neurogenesis in stress-induced models^90^ and abstinence following alcohol drinking^91^. Here, the treatment with DES was able to increase neurogenesis in the ventral hippocampus of both control and social isolated mice.

In the context of SI, we propose that the neurogenic niche determines whether the depressive phenotype and the social memory deficit are connected or not. As suggested before, and supported by our results, affective behaviors are modulated by the neurogenesis levels in the vHIP. On the other hand, we suggest that social memory is particularly sensitive to neurogenesis in the dHIP, compared to vHIP and OB^40^. In fact, neither fluoxetine or desipramine substantially affected neurogenesis in the dHIP.

The unique experimental condition wherein social memory deficit and depressive behavior caused by SI were recovered was after chronic treatment with fluoxetine. Thus, we predicted that the region wherein the fluoxetine-dependent increase in neurogenesis was higher is the region with a crucial role in both cognitive and emotional impairments, caused by ST. Therefore, our results support a pivotal role for the OB. The percentage increase in neurogenesis caused by FLX was about two times higher in the OB, compared to dHIP and vHIP. Nevertheless, one could argue that OB neurogenesis was also more affected by DES, compared to other neural substrates, although desipramine did not improve social memory. However, it is worth noticing that the magnitude of increase was of about 50% and 100% after DES and FLX, respectively. Furthermore, there was no region-dependent effect of FLX or DES in the control group, suggesting that the antidepressants efficiency on increasing neurogenesis may be sensitive to the environment where the animal lives. Interestingly, a recent study showed that the efficiency of FLX on rescuing the depressive phenotype caused by chronic stress depends on whether the animal is kept in enriched environment or not during the treatment^90^.

Compared to the hippocampus, there are only few studies showing that FLX increases neurogenesis in the OB^87^, despite its densely serotoninergic innervation from the raphe nuclei^92^. Equally less investigated is the function of serotoninergic terminals in the OB. It was showed that the stimulation of raphe nuclei regulates odor inputs in the OB^93^ and improves pattern separation of odors in mice^94^. Consistent with our results, corticosterone treated-mice are depressed, displaying olfactory memory deficit and decreased OB-neurogenesis, and all these effects were recovered by chronic FLX treatment^87^.

Hippocampus sends direct excitatory inputs to the olfactory system^95,96^ and this top-down modulation is important to predict, expect, and retrieve memories from previous experiences^97–99^. However, such projections are not equally distributed along the anterior–posterior tract. For example, pyramidal neurons from the ventral hippocampus project massively to medial anterior olfactory nucleus, while dorsal innervate increasingly more lateral positions^100^. We showed recently that OB theta oscillations drive dorsal hippocampus gamma amplitude during long-term social memory retrieval. This OB–dHIP coupling was impaired in SI mice during memory retrieval^37^. Taken together, we may speculate that FLX has recovered the social memory deficit by reinstating OB–dHIP coupling, though a new study is needed to test this hypothesis.

Finally, we tested whether enriched environment would be as effective as the fluoxetine and desipramine on rescuing SI mice depressive-like behavior. Our results showed a consistent antidepressant effect of enriched environment. As we already demonstrated a cause–effect relationship between social memory and neurogenesis in SI mice^38^, we may suggest that neurogenesis is possibly playing a role on the antidepressant effect of enriched environment as well.
